# Effects of Mitophagy on Regulatory T Cell Function in Patients With Myasthenia Gravis

**DOI:** 10.3389/fneur.2020.00238

**Published:** 2020-04-07

**Authors:** Na Wang, Jiang Yuan, Md Rezaul Karim, Ping Zhong, Yan-Peng Sun, Hong-Yan Zhang, Yun-Fu Wang

**Affiliations:** ^1^Department of Neurology, Taihe Hospital of Hubei University of Medicine, Shiyan, China; ^2^Biomedical Research Institute of Hubei University of Medicine, Shiyan, China; ^3^Department of Preventive Medicine, Hubei University of Medicine, Shiyan, China

**Keywords:** myasthenia gravis, mitophagy, regulatory T cell, rapamycin, 3-methyladenine

## Abstract

**Objective:** This study was conducted to determine whether regulatory T cells (CD4^+^CD25^+^T, Tregs) show abnormal mitophagy as well as the function of Tregs in patients with myasthenia gravis (MG).

**Methods:** CD4^+^T cells and CD4^+^CD25^+^Treg cells were obtained from 15 patients with MG (MG group) and 15 controls (N group). Tregs from the MG group were subjected to rapamycin-induced culture for 48 h (Rapa group) and 3-methyladenine-induced culture for 48 h (3-MA group). The levels of mitophagy in Tregs were then observed through electron and confocal microscopy. Expression of the autophagy-related protein LC3-II was detected by western blotting, and mitochondrial function in each group was evaluated by flow cytometry. Inhibition of Treg cell proliferation was detected by flow cytometry.

**Results:** Mitophagy in the MG group was lower than that in the N group; it was higher in the Rapa group compared to that in the MG group and lowered in the 3-MA group than in the MG group. Expression of the autophagy-related protein LC3-II was lower in the MG group than in the N group, higher in the Rapa group than in the MG group, and lower in the 3-MA group than in the MG group. The mitochondrial membrane potential was lower in the MG group compared to that in the N group; it was higher in the Rapa group than in the MG group and lowered in the 3-MA group than in the MG group. Inhibition of Treg proliferation was lower in the MG group than in the N group; it was higher in the Rapa group than in the MG group and lowered in the 3-MA group than in the MG group.

**Conclusion:** The decreased mitochondrial membrane potential and mitophagy in Tregs in the MG group may be related to a decreased inhibition of Treg proliferation. The mitochondrial membrane potential was increased after adding the autophagy agent Rapa to enhance mitophagy, and the proliferation inhibition function of Tregs was also enhanced. The autophagy agent 3-MA down-regulated mitophagy, which decreased the mitochondrial membrane potential and inhibitory effect of Tregs. These results reveal the possible cellular immune mechanism of Treg dysfunction in MG.

## Introduction

The acetylcholine receptor (AChR) antibody is the main mediator of cellular immune dependence and complement involvement in myasthenia gravis (MG). In AChR-related acquired autoimmune diseases, the receptor primarily involves the neuromuscular junction of the postsynaptic membrane. These diseases often manifest as a partial or full body sickness of the skeletal muscle. Muscle-specific tyrosine kinase (MuSK) receptor, low-density lipoprotein receptor-related protein 4 (LRP4) receptor, titin receptor (Titin R), and ryanodine receptor (RyR) are the few mediators in MG. Still, the clinical disease, immunopathogenesis, endplate pathology, treatments, and therapeutic targets differ (1, 2). In the present treatment options in MG, there are many ways to relieve symptoms and reduce the recurrence of the disease, such as cholinesterase inhibitors, glucocorticoids, immunosuppressives, anti CD20 monoclonal antibody, plasma exchange, IVIg and thymus excision therapy. All these treatment options share their unspecific mechanism, unable to cure; thus, illness aggravating, or recurrence may remain. Most of the patients need long-term therapy to maintain improvement at an effective dose of immunosuppressants (3, 4), or there may be side effects such as bone marrow depression, kidney damage, femoral head necrosis, etc. Some drugs are expensive and may not be affordable to many patients, such as rituxan or eculizumab (2, 5). Therefore, it is important to develop new methods to treat and prevent the development of MG disease by inhibiting pathological changes in the immune system.

In AChR-positive MG, the production of autoantibodies by pathogenic B cells are T cell-dependent (2). During the immune response, CD4^+^T cells are activated, proliferated, and finally differentiate into Th1, Th2, Th17, and Treg cells, which mediate cellular and humoral immune responses. Th1 and Th2 cells regulate each other, Th1 and Th2 cells regulate Th17 cells, and Treg cells regulate Th17 cells, etc., so that immune effects and immunosuppression are in a delicate and complex balance. Among them, Treg cells have the functions of inhibiting the activation of autoreactive T cells and autoreactive B cells and play a major role in the regulation of the immune response and autoimmunity (6). In autoimmune diseases like MG, it is the imbalance between Th1 and Th2 cells and/or between Treg cells and Th17 cells (7). At present, it is believed that the abnormal number or function of CD4^+^CD25^+^Treg may be the trigger point for MG (8). Therefore, in this study, we chose to start with Treg cells as the research object. Many studies have confirmed that regulatory T cells are involved in MG pathogenesis. The number of CD4^+^CD25^+^Foxp3^+^Tregs and the protein expression of Foxp3 in the peripheral blood of MG patients were decreased (9). Xu et al. (10) demonstrated that the number of CD4^+^CD25^+^Foxp3^+^Tregs in MG patients was lower than that in controls. It has been reported (11) that there are lower Treg numbers and dysfunction in both experimental MG model and MG patients, it can be adjusted in a variety of ways to regulate Treg function and number, which can improve the disease condition. These reports suggest that the abnormal Treg cells are involved in MG pathogenesis.

Mitophagy has been observed as selective autophagy in various physiological processes and diseases, such as reticulocyte development (12). Geisler et al. (13) studied mitophagy and its molecular mechanism of Parkinson's disease. Mitophagy is closely related to the development, activation, and differentiation of T cells. Michalek et al. (14) found that different metabolic states determine the differentiation direction of CD4^+^T cells. Treg differentiation is dependent on mitochondrial lipid oxidation, while effector T cells are more dependent on glycolysis. Mitophagy also plays an essential role in the development and maturation of T lymphocytes (15). It has been shown that in VPS34 or Atg7 knockout mice, the mitophagy of CD4^+^T cells was decreased, while intracellular mitochondrial clearance dysfunction and reactive oxygen species (ROS) production were increased. These events lead to T cell dysfunction or apoptosis; thus, T cell metabolic states are closely related to the intracellular mitochondrial quality and quantity of stable T cells (16, 17).

The functional defects of Tregs in patients with MG may be closely related to their mitophagic abnormalities. The mitophagy function of Tregs, excess or damaged mitochondrial accumulation in cells, energy metabolism disorders, abnormal intracellular environment, activation of Tregs, proliferation disorders, and autoimmune tolerance of the body lead to the occurrence and progression of MG. To confirm this hypothesis, the relationship between mitophagy and its function in Tregs in MG were used to identify whether mitophagy occurred in Tregs in the peripheral blood of patients with MG. The mitophagy state of Tregs was regulated by 3-methyladenine (3-MA) and rapamycin (Rapa) *in vitro* to observe the effect of differences in mitophagy on Treg function.

## Materials and Methods

### Specimen Collection

In this study, all samples, including those from patients with MG and controls, were collected from the department of neurology, Taihe Hospital of Hubei University of Medicine, China. The samples were divided into two groups: (1) MG group: 15 patients (5 males and 10 females), according to the Osserman type, 9 cases of type IIA, and 6 cases of type IIB, with a mean patient age of 35.9 ± 11.2 years (see Table 1). (2) Control group: 15 healthy volunteers, age(s), and sex(s) were matched to the MG group, with a mean age of 36.1 ± 9.5 years.

**Table 1:** Specimen collection of patients with MG.

**Serial no**.	**Age (years)**	**Sex (M/F)**	**Osserman type**	**Disease Duration**	**Antibodies**	**Thymus**	**Other autoimmune diseases**
01	39	F	IIA	2 months	AChR	Thymoma (MRI)	–
02	25	F	IIB	2 months	AChR	Thymic hyperplasia (CT)	Idiopathic thrombocyto- penic purpura (ITTP)
03	48	M	IIA	10 months	AChR	Normal (CT)	–
04	23	F	IIA	2 months	AChR	Thymoma (CT)	Connective tissue diseases
05	45	M	IIA	10 days	AChR	Thymic hyperplasia (CT)	
06	45	F	IIB	1 year	AChR	Thymoma (CT), B2/B3 (Pathology)	–
07	47	F	IIB	1 month	AChR	Normal (MRI)	Idiopathic thrombocyto- penic purpura (ITTP)
08	28	F	IIA	3 years	AChR	Normal (CT)	Undifferentiated connective tissue diseases
09	43	M	IIB	6 months	AChR	Thymoma (CT), B2 (Pathology)	–
10	41	M	IIA	4 months	AChR	Thymoma (CT)	Spinal cord demyelinating disease
11	44	F	IIA	4 months	AChR	Thymoma (CT), B2 (Pathology)	–
12	46	M	IIB	10 days	AChR	Thymoma (CT), AB (Pathology)	–
13	40	F	IIA	2 days	AChR	Thymic hyperplasia (CT)	–
14	38	F	IIB	3 years	AChR, Titin R	Normal (MRI)	–
15	45	F	IIA	1 week	AChR	Thymoma (CT), B2 /B3 (Pathology)	–

“*–”, not found with other immune-related diseases*.

Subject inclusion criteria were as follows: (1) volunteers with following medical history were excluded: acute or chronic liver disease, acute or chronic kidney disease, coronary syndrome, various infectious or other immune diseases, valvular heart disease, malignant tumor, diabetes, major surgery, or severe trauma, (2) MG group: after admission, the patients were diagnosed with MG by clinical diagnosis, and all patients were Osserman type II, all the AChR-positive. These patients had not been administered anticholinergic and hormonal drugs within two weeks before the visit, and (3) Control group: healthy volunteers without the disease, was not administered anticholinergics, steroids, or other specific drugs for the past two years. In the hospital, no MG patients had been administered anticholinergics or steroid drugs before collecting a sample. Venous blood (30 mL) was collected with sterile anticoagulant syringes under aseptic precautions from the brachial vein. All volunteers were informed in detail before the test specific implementation process and provided consent. The hospital ethics committee approved the study.

### CD4^+^CD25^+^Treg Separation and Drug Treatment

Peripheral blood mononuclear cells (PBMCs) were obtained by ficoll gradient centrifugation. After removing the supernatant, the cells were counted (3–4 × 10^7^ cells/mL) using a hemocytometer. CD4^+^T cells (5–7 ×10^6^ cells/mL) and CD4^+^CD25^+^Treg cells (2–5 × 10^5^ cells/mL) were obtained using an LD column, MS column, and magnetic bead sorting kit (MiltenyiBiotec, Bergisch Gladbach, Germany). CD4-FITC, CD25-PE, CD4-FITC, and CD25-PE antibodies were incubated with CD4^+^CD25^+^T cells, and the purity of CD4^+^T and CD4^+^CD25^+^T cells was measured by flow cytometry (BD Biosciences, Franklin Lakes, NJ, USA). The purity of CD4^+^T cells was more than 95%, while that of CD4^+^CD25^+^T cells was 90.2 ± 2.6%.

A 1 mL culture solution was prepared with 50 nM Rapa (Sigma, St. Louis, MO, USA) and 2.5 mM 3-MA (Sigma) in 1 mL of culture medium (Roswell Park Memorial Institute (RPMI) 1640 + 10% fetal bovine serum (FBS) + penicillin + streptomycin; Gibco, Grand Island, NY, USA) and incubated at 37°C for 15–30 min. Another 2 mL of culture solution was prepared and incubated at 37°C for 15–30 min. After obtaining 1 mL of culture solution, Tregs were divided into four groups: MG group1, MG group 2, MG group 3, and normal group (1 × 10^5^ cells/mL/group). For cell culture, the normal group was mixed with 1 mL culture solution (as described above), MG group 1 was mixed with alternative 1 mL culture solution, MG group 2 was mixed with 1 mL Rapa-containing culture solution, and MG group 3 was mixed with 1 mL 3-MA-containing culture solution. After 48 h of incubation at 37°C, the cells were collected and prepared for subsequent experiments.

In the following tests, the research team included personnel responsible for the recording of the film and data for subsequent experiments. The researchers were blinded to the sample groupings.

### Transmission Electron Microscopy

CD4^+^CD25^+^Tregs from each group were treated with 2.5% glutaraldehyde (diluted with pH 7.4; 0.1 mmol/L phosphate buffer) at 4°C for 2–4 h and then rinsed with 0.1 mmol/L phosphate buffer for 15 min three times. The cells were incubated in 1% osmium acid (diluted with pH 7.4; 0.1 mmol/L phosphate buffer) for that room temperature (20°C) and then rinsed three times with 0.1 mmol/L phosphate buffer (pH7.4) for 2 h for 15 min each time. Subsequently, 50, 70, 80, 90, 95, and 100% alcohol gradients were used to dehydrate the samples for 15 min; 812 embedding agents were added to penetrate the cells overnight, followed by polymerization for 48 h at 60°C. Next, 60–80 nM ultra-thin slices were prepared, after which uranium-lead double staining (2% uranium-saturated aqueous solution, lead citrate, and buffer for 15 min) and drying at room temperature were performed. The total number of autophagic cells in each group (Hitachi, Tokyo, Japan) was observed with an electron microscope for five slides in each group. Then each slide was randomly chosen to calculate the total number of autophagic cells. Finally, the average number of autophagic cells in each section was analyzed statistically. The early stage of mitochondrial autophagy was distinguished based on the characteristics of mitochondria, such as bilayer membrane and cristae, and its fusion with the lysosome could be roughly recognized by a monolayer membrane or digested residue (18).

### Laser Confocal Microscopy

Lysotracker and Mitotracker probe solutions (400 nM; catalog numbers L7528 and M7514; Life Technologies, Carlsbad, CA, USA) were incubated with the cells at 37°C for 30 min. All four groups of Treg cultures were collected into four different 15 mL centrifuge tubes and then centrifuged, after which the supernatant was discarded. After washing with phosphate-buffered saline (PBS), 1 mL of pre-warm Lysotracker and Mitotracker medium was added, mixed gently, and transferred into a cell incubator. After incubation for 30 min, the supernatants were removed by centrifugation. The samples were washed twice with PBS, and 50 μL of the medium was added to resuspend the cells by thorough mixing. Next, 100 μL medium was placed on a slide and covered with coverslips for immediate observation under a confocal microscope (Germa company, Germany) under the film. The fusion ratio of the two different color probes was statistically analyzed. Under a confocal laser scanning confocal microscope, the lysosomes showed red fluorescence, and mitochondria showed green fluorescence. Co-localization appeared as orange fluorescence. Under the same objective lens, the ratio of fused cell numbers to total cell numbers were calculated and statistically analyzed (i.e., ratio of orange fluorescent cells to total cells).

### Expression of Autophagy-Related Protein LC3-II Detected by Western Blotting

After collecting Tregs from each group (*n* = 15/group), total protein was extracted, and the protein concentration was measured by the bicinchoninic acid method. Sodium dodecyl sulfate-polyacrylamide gel electrophoresis was performed for ~1.5 h. Then, proteins were transferred to a nitrocellulose membrane using a semi-dry method for ~50 min. After the transfer, 5% bovine serum albumin was incubated with the membrane at room temperature on a shaker for 30 min and then incubated overnight with LC3 antibody (Cell Signaling Technology, Danvers, MA, USA) and rabbit polyclonal glyceraldehyde-3-phosphate dehydrogenase (GAPDH) antibody (Hangzhou, China). Tris-buffered saline containing Tween 20 was incubated with horseradish peroxidase-labeled goat anti-rabbit IgG (Cell Signaling Technology) for 1 h at room temperature (20–25°C); electrochemiluminescence detection was performed to evaluate the protein signal by exposure to a gel imager. Protein levels were normalized to GAPDH, and changes were determined such that the LC3-II/GAPDH ratio reflected the degree of autophagic protein expression. LC3-II is more hydrophobic than GADPH, and thus LC3-I appeared as the upper band in the electropherogram, while LC3-II was in the lower band. The ratio of LC3-II/GAPDH was calculated based on GAPDH as the internal reference level of expression for statistical analysis.

### Mitochondrial Membrane Potential of Tregs Detected by Flow Cytometry

Tregs in each group (*n* = 15/group) were collected and centrifuged. The cells were resuspended in 0.5 mL RPMI1640 medium (Gibco) and mixed with 10 μg/mL JC-10 mitochondrial membrane potential fluorescent probe (Solarbio, Beijing, China). After incubation for 20 min, the cells were washed twice with PBS and resuspended in PBS. Flow cytometry was performed immediately to detect the cells. JC-10 mainly aggregates to form a polymer in the mitochondrial matrix is the red fluorescence, when the membrane potential declined in the form of the monomer for the green fluorescence. The change in the green fluorescence ratio in this experiment indicated changes in the membrane potential.

### Treg Ability to Inhibit CD4^+^T Cell Proliferation Detected by Flow Cytometry

CD4^+^T control group cells were isolated and resuspended in 1 mL of culture medium (without FBS). After adding 5 μL/mL carboxyl fluoresce in succinimide ester (CFSE; Beyotime, Shanghai, China) and incubation at 37°C in the dark for 10 min, 1 mL FBS (Gibco) was added to stop the reaction for 10 min. The samples were washed twice with PBS and resuspended in complete medium (RPMI1640 medium + 10% FBS + penicillin, streptomycin). CD3, CD28, and IL-2 (R&D Systems, Minneapolis, MN, USA), and CD4^+^CD25^+^Tregsfrom each group (*n* = 15/group) were added to the CD4^+^CD25^+^T: CD4^+^T = 1:1 cells for 4 days, followed by washing twice with PBS. Flow cytometry was performed, and the average fluorescence intensity of CFSE was detected using FLOWJO software (as CD4^+^T cells proliferated, the average fluorescence intensity decreased, and thus the average fluorescence intensity is directly proportional to the inhibition of Treg proliferation) (19–21).

### Data Analysis and Statistics

All data were expressed as the mean ± standard deviation (*x* ± *s*). SPSS V17.0 software (SPSS, Inc., Chicago, IL, USA) was performed to analyze the data, and analysis of variance was performed to compare multiple samples. ^*^P < 0.05 was considered as statistically significant.

## Results

### Electron Microscopy

**Figure 1:**
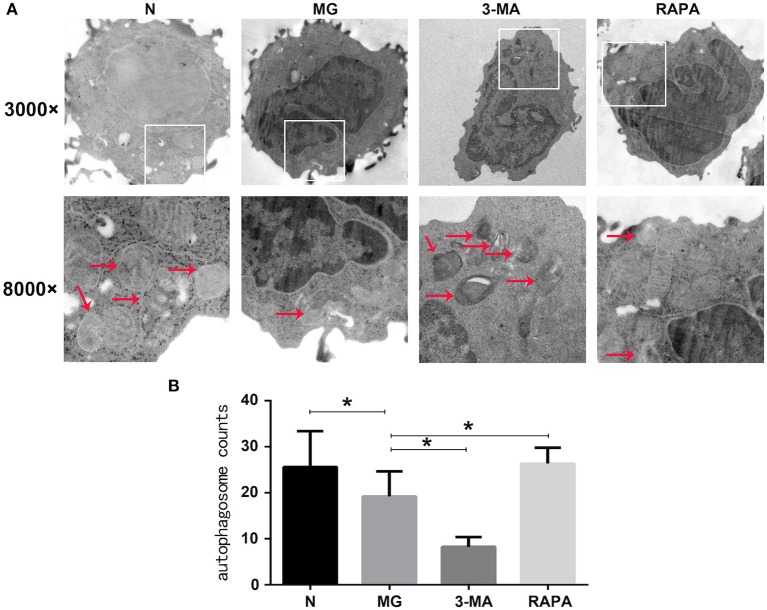
Mitophagy under electron microscopy. **(A)** Mitophagy of healthy control group (N), myasthenia gravis (MG), 3-methyladenine treatment group (3-MA), and rapamycin treatment group (Rapa). In the first row is 3000×, the second row is 8000×. **(B)** N, MG, Rapa, and 3-MA four groups of statistical analyses (*P < 0.05).

Autophagosomes and mitochondria of CD4^+^CD25^+^Tregs were observed by electron microscopy in cells from the control group, MG group, Rapa group, and 3-MA group of patients with MG. A bilayer membrane-like structure containing cytosolic components was observed (19.20 ± 5.49, a sum of structures in five randomly selected cells); the cytoplasmic components were mainly mitochondrial fragments. Compared to the control group (25.60 ± 7.81), the observed rate of autophagosome-like structures was significantly reduced (*P* < 0.05). Mitochondrial autophagy was higher in the Rapa group than in the MG group (26.33 ± 3.50, *P* < 0.05). Mitochondrial autophagy was lower in the 3-MG group than in the MG group (8.27 ± 2.12, *P* < 0.05; see Figure 1).

### Laser Confocal Microscopy

**Figure 2:**
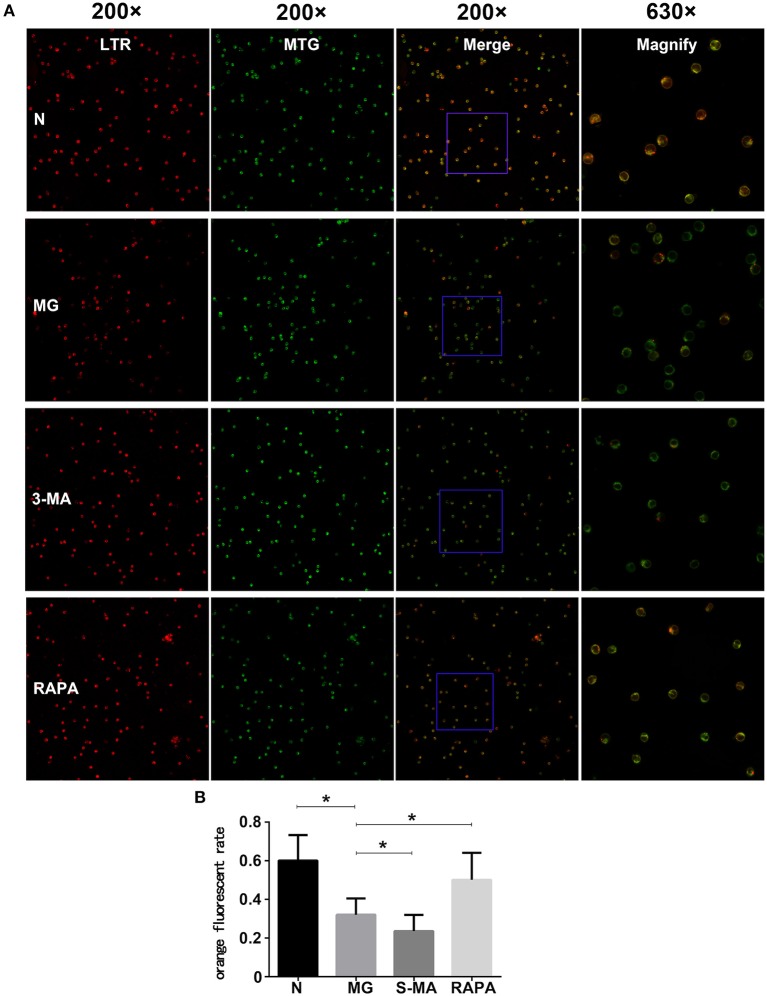
Treg cell fusion of lysosomes and mitochondria. **(A)** The Treg cells fluorescent pictures of four groups in confocal microscopy. Each group from left to right are fluorescence LTR, MTR, and fusion display (200×), as the rightmost fused fluorescent randomly selected partially enlarged (630×). **(B)** Results of the statistical analysis of the four groups (*P < 0.05).

Each Treg group was subjected to laser confocal microscopy using Lysotracker and Mitotracker. Through laser confocal microscopy, mitochondria showed green fluorescence, lysosomes showed red fluorescence, and mitochondrion and lysosome colocalized autophagosomes showed orange (or yellow) fluorescence. The results showed that the ratio of the number of orange (or yellow) fluorescent cells to the total number of cells in the MG group (0.321 ± 0.085) was significantly lower than that of the control group (0.603 ± 0.133, *P* < 0.05). The ratio of orange (or yellow) fluorescent cells in the Rapa group was significantly increased (0.495 ± 0.139, *P* < 0.05), and the ratio in the 3-MA group (0.237 ± 0.828) was decreased (*P* < 0.05). This suggests that patients with MG can have maturation disorders of mitochondrial autophagosomes, which were improved after Rapa treatment and were further aggravated after 3-MA treatment (see Figure 2).

### Autophagy Protein LC3-II Expression Levels

**Figure 3:**
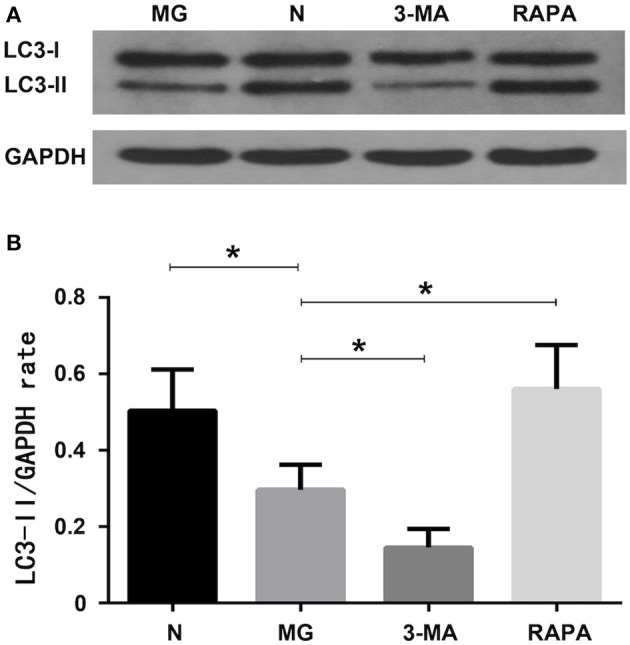
Autophagy-related protein expression of LC3-II. **(A)** Western blot detection findings; **(B)** Statistical analysis of the four groups (*P < 0.05).

Expression of the autophagy protein LC3-II was significantly decreased (*P* < 0.05) in the MG group (0.297 ± 0.065) compared to that in the normal group (0.504 ± 0.108) as detected by western blotting. The Rapa group was increased (0.561 ± 0.115, *P* < 0.05), while the 3-MA group was decreased (0.146 ± 0.490, *P* < 0.05) compared to the MG group. This suggests that autophagy in the MG group was lower than that in the control group, autophagy was increased after treatment with rapamycin but decreased after treatment with 3-MA (see Figure 3).

### Treg Mitochondrial Membrane Potential

**Figure 4:**
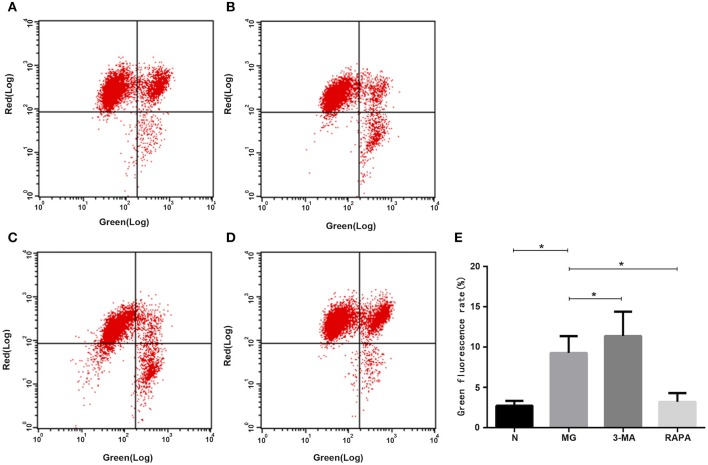
Mitochondrial membrane potential. In the flow cytometry images, G indicates green fluorescence, R indicates red fluorescence. Mitochondrial membrane potential in the **(A)** healthy control group, **(B)** MG group, **(C)**, 3-MA pretreatment group, **(D)** Rapa pretreatment group, and **(E)** Statistical analysis results are shown as (*P < 0.05).

JC-10 labeling for flow cytometry analysis showed that when the mitochondrial membrane potential was high, JC-10 accumulated in the mitochondrial matrix to form a polymer, which produced red fluorescence. When the mitochondrial membrane potential was low, JC-10 did not aggregate in the mitochondrial matrix to form monomer and produced green fluorescence. The green fluorescence intensity ratio was significantly higher in the MG group (9.28 ± 2.09%) than in the control group (2.73 ± 0.617%) and, thus, the membrane potential was significantly decreased (*P* < 0.05). Rapa group (3.21 ± 1.09%) than MG ratio decreased, membrane potential increased (*P* < 0.05), 3-MA group (11.37 ± 3.02%) than MG ratio increased, membrane potential decreased (*P* < 0.05). These results suggest that the mitochondrial membrane potential changes with changes in the autophagy state (see Figure 4).

### Inhibition of Treg Proliferation

**Figure 5:**
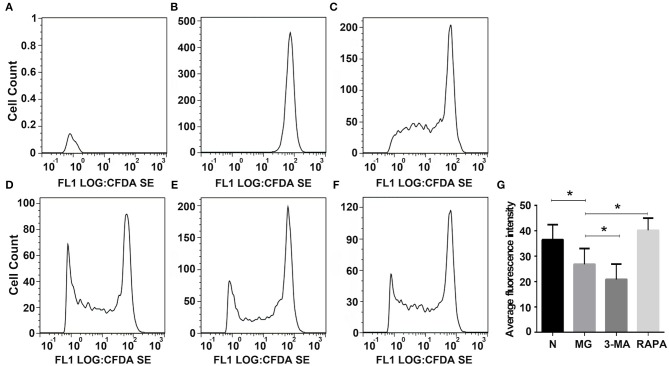
Inhibition of Tregson the proliferation of normal CD4^+^T cells. FLOWJO software was used to analyze the flow cytometry pictures. **(A)** Autofluorescence, **(B)** not give any irritation, **(C)** control group added Treg cells were co-cultured, **(D)** 3-MA pretreated Treg cells were co-cultured, **(E)** Rapa pretreated Treg cells were co-culture, **(F)** MG group Treg cells were co-cultured, and **(G)** Statistical analysis of each group (*P < 0.05).

To evaluate the inhibition of the proliferation of normal CD4^+^T cells by Tregs, Tregs were co-cultured with CFSE-labeled normal CD4^+^T cells, and the proliferation ability of CD4^+^T cells was detected by flow cytometry. CFSE released green fluorescence after covalently binding to intracellular proteins in living cells. During cell division and proliferation, fluorescence can be evenly distributed to two daughter cells, as cells proliferate, the fluorescence intensity gradually weakens, indicating a weaker ability of Tregs to inhibit proliferation. Proliferation was significantly lower in the MG group (26.82 ± 6.25) than in the normal group (36.49 ± 5.94, *P* < 0.05); it was higher in the Rapa group (40.18 ± 4.82) than in the MG group (*P* < 0.05) and lower in the 3-MA group (20.81 ± 6.13) than in the MG group (*P* < 0.05; see Figure 5).

## Discussion

It is well-known that MG is an autoimmune disease, but the mechanisms initiation of its immune response remains unclear. There are a large number of activated autoreactive CD4^+^T cells in the thymus, particularly, AChR-reactive CD4^+^T cells are involved in MG. Numerous studies have shown that the number and function of Tregs are critical factors involved in the pathogenesis of MG (9–11).

In this study, peripheral blood Tregs were used to investigate the relationship between MG and mitophagy. Mitophagy is involved in various stages of T cell development and differentiation (22). Mitophagy is a type of intracellular anti-oxidative protection mechanism in activated human T cells, which maintains normal mitochondrial autophagy function, removes excess or damaged mitochondria in a timely manner. It also maintains normal energy metabolism and forms the basis for maintaining T cell homeostasis and healthy immune function (23, 24). Stephenson et al. (25) revealed an abnormal increase in mitochondrial volume in peripheral blood T cells lacking autophagy-associated protein 5 (Atg5), suggesting that autophagy plays a crucial role in mitochondrial maintenance and T cell survival. Watanabe et al. (26) found that autophagy-deficient CD4^+^T cells were more likely to induce apoptosis through the apoptotic pathway. In contrast, autophagy-deficient T-cell apoptosis occurred because of mitochondrial autophagy damage, leading to ROS accumulation and activation. Blocking autophagy in T cells increases the intracellular levels of mitochondria and ROS. Wei et al. (27) and Kabat et al. (28) showed that autophagy plays a critical role in regulating Tregs, suggesting that defects in autophagy decrease survival and lineage stability of peripheral blood Tregs. These studies indicate that mitophagy is essential for maintaining the number and function of Tregs. This study provides a theoretical basis for the relationship between MG and mitophagy. It has been reported that (29) peripheral blood T cells contain fewer mitochondria than thymic T cells; thus, mitochondrial degradation is more dependent on autophagy. Autophagy is reduced when the number of abnormal mitochondria is increased; elevated ROS in T cells leads to cell damage that causes disease. Abnormal mitophagy of peripheral blood Tregs may have a greater impact than thymic Treg cells on the pathogenesis of MG.

Additionally, many autoimmune diseases, such as systemic lupus erythematosus, Crohn's disease, diabetes, congenital pulmonary fibrosis, and MG, are considered as being closely related to abnormal mitophagy (30–32). MG may, thus, be related to mitophagy. In this study, we extracted Tregs from the peripheral blood of patients with MG. We then performed electron microscopy and laser confocal microscopy (33) to qualitatively evaluate Treg mitophagy in these cells and compare to that in cells of the control group, which was significantly decreased (Figures 1, 2). LC3-II expression in the peripheral blood Tregs of patients with MG was reduced (Figure 3), indicating that the autophagy level was reduced, consistent with the qualitative test. The mitochondrial membrane potential of Tregs was detected by flow cytometry using the JC-10 probe. The mitochondrial membrane potential of Tregs in the MG group was significantly lower than that in the normal group (Figures 4A,B), destroying the mitochondrial integrity and permeability of Tregs. Intracellular mitochondrial morphological abnormalities that cannot be cleared by mitophagy result in high levels of ROS and mitochondrial toxic substances, causing the peripheral blood Tregs in MG patients to decrease or exhibit dysfunction. Finally, CD4^+^T cell proliferation of Treg cells in patients with MG was significantly inhibited compared to that in the control group (Figures 5C,F). Previous studies (34–37) also showed a decrease in the inhibition of the proliferation of Tregs in MG patients, but the causes of abnormal cell function remained unclear, suggesting that Treg dysfunction is associated with reduced mitophagy.

3-MA and Rapa were used to regulate the autophagy of Tregs with MG. The MG group was used as a control, Rapa increased mitophagy, whereas 3-MA decreased mitophagy (Figure 1). Co-localization of LTR acidic lysosomes and MTG mitochondria was higher in the MG group and lowered in the 3-MA group (Figure 2). LC3-II expression in peripheral blood Tregs of the Rapa group was much higher than in the MG group, indicating that Rapa increases autophagy, while the 3-MA group showed lower expression of LC3-II compared to the MG group (Figure 3), indicating decreased autophagy. Finally, we detected the mitochondrial membrane potential of Tregs and inhibition of proliferation by Tregs in the Rapa and 3-MA groups. Rapa increased the mitochondrial membrane potential of Tregs (Figure 4D), altered the intracellular mitochondrial morphology, increased the number and function of Tregs, and, thus, enhanced the immunoproliferative ability of Tregs (Figure 5E); 3-MA caused the opposite results (Figures 4C, 5D).

Therefore, we verified that in patients with MG, peripheral blood Tregs were increased because of reduced mitophagy. Additionally, the different mitophagy state of Tregs has a noticeable effect on the status of mitochondria and the function of Tregs. That was confirmed related to decreasing in the mitophagy level on Tregs in the peripheral blood of patients with MG.

MG is mainly treated with anti-cholinesterase drugs, immunotherapy, plasma exchange, intravenous immunoglobulin (IVIg), or thymus extraction and requires the long-term use of immunosuppressants. High-dose glucocorticoids alleviate the condition, but also cause various complications such as femoral head necrosis, osteoporosis, increased appetite, weight gain, centripetal obesity, hypertension, high blood glucose, cataracts, glaucoma, endocrine disorders, mental disorders, gastrointestinal symptoms, etc. Therefore, more effective and less reactive treatments should be identified to study the pathogenesis of MG.

Rapa (sirolimus) is an immunological preparation whose safety and effectiveness have been demonstrated. Its derivatives, such as everolimus and tacrolimus, are applied to tumors, immune diseases, and epilepsy, among other conditions. Inhibiting the mTOR pathway clearly improves the symptoms of rheumatoid arthritis, multiple sclerosis, autoimmune encephalomyelitis, and other animal models (38, 39). In a patient with MG complicated with iatrogenic Kaposi sarcoma, the tumor was cured, and the AChR antibody concentration was reduced after treatment with Rapa (40). Additionally, previous studies showed that Rapa effectively treated malignant thymoma (41).

Similarly, Rapa significantly improved symptoms in MG animal model rats (42). The mortality of rats after Rapa treatment was lower than that in rats administered the traditional immunosuppressant cyclophosphamide. In recent years, many studies (43–45) have shown that tacrolimus is important for treating MG, particularly for reducing the use of glucocorticoids, and is relatively safe. Our results suggest that may be Rapa intervention is a better treatment option for patients with MG or the other drugs, which can raise the mitophagy in Treg cell; this requires further study of larger sample size.

In this study, treatment of Tregs with Rapa increased autophagy levels, after which the function of Tregs returned to nearly normal levels, suggesting that the mTOR inhibitor Rapa can be used to treat patients with MG, but further studies are needed to determine the underlying mechanisms.

## Conclusion

The mitophagy and function of CD4^+^CD25^+^Treg cells in patients with MG were decreased. The autophagy state of CD4^+^CD25^+^Treg cells in the peripheral blood of patients with MG was adjusted *in vitro* to observe changes in the immunoregulatory role of these cells. The mitophagy of CD4^+^CD25^+^Treg cells may be closely related to their function, suggesting a possible mechanism for the effects of CD4^+^CD25^+^Treg cells on dysfunction in patients with MG.

## Data Availability Statement

The datasets generated for this study are available on request to the corresponding author.

## Ethics Statement

All subjects were informed in detail before the test specific implementation process and signed a written form of informed consent after a verbal agreement to participate in the study. The study protocols were approved by the hospital ethics committee, Taihe Hospital of Hubei University of Medicine, and institutional review board (IRB), Hubei University of Medicine, Shiyan, Hubei, China.

## Consent for Publication

All the authors have agreed to publish this work.

## Author Contributions

All authors listed have made a substantial, direct and intellectual contribution to the work, and approved it for publication.

### Conflict of Interest

The authors declare that the research was conducted in the absence of any commercial or financial relationships that could be construed as a potential conflict of interest.

## Abbreviations

Tregs: Regulatory T cells
ROS: Reactive oxygen species
MG: Myasthenia gravis
RPMI: Roswell Park Memorial Institute
N: Normal
CFSE: Carboxyl fluorescein succinimide ester
Rapa: Rapamycin
FBS: Fetal bovine serum
3-MA: 3-methyladenine
mTOR: Mechanistic target of rapamycin
AChR: Acetylcholine receptor
LC3-II: Microtubule-associated protein1 light chain 3
GAPDH: Glyceraldehyde-3-phosphate dehydrogenase
MuSK: Muscle-specific tyrosine kinase
Titin R: Titin receptor
LRP4: low-density lipoprotein receptor-related protein 4
RyR: Ryanodine Receptor
PBMC: Peripheral blood mononuclear cell.
